# Early-Age Behaviour of Calcium Sulfoaluminate Cement Blended with Fly Ash-Containing Portland Cement: Hydration, Workability and Strength Development

**DOI:** 10.3390/ma19214530

**Published:** 2026-10-27

**Authors:** Sergio Ruiz-Martínez, Thanyarat Buasiri, Lisa Burris, Andrzej Cwirzen

**Affiliations:** 1 Building Materials Group, Department of Civil, Environmental and Natural Resources Engineering, Luleå University of Technology, 97187 Lulea, Sweden; thanyarat.buasiri@ltu.se (T.B.); andrzej.cwirzen@ltu.se (A.C.); 2 Department of Civil, Environmental and Geodetic Engineering, The Ohio State University, 70 Hitchcock Hall, 2070 Neil Ave, Columbus, OH 43210, USA; burris.189@osu.edu

**Keywords:** calcium sulfoaluminate cement, water-to-binder ratio, workability window, early age strength, hydration

## Abstract

Calcium sulfoaluminate cement (CSA) offers potential performance and environmental benefits to cementitious mixes. Regardless, the joint behavior of CSA cement together with fly ash blended portland cement (PC) remains insufficiently understood. Therefore, this document presents an experimental investigation of the effect of simultaneous variations of CSA as PC replacement and water to binder (w/b) ratios on the hydration, fresh state, and early-age mechanical properties of paste and mortar mixes. Laboratory tests were based on statistical experimental design with CSA percentages varying from 25 to 75% and w/b ratio from 0.35 to 0.52. Findings indicate that increasing the CSA content significantly reduces the setting time while w/b ratio variations result in minimal effects. Hybrid mixes exhibited slump and slump retention time (workability window) reductions as CSA content increased. Compressive and flexural strength decreased with higher w/b ratio, but CSA cement content increments promoted compressive strength with time while flexural strength evolution was affected by higher CSA cement content. Among the investigated compositions, the mixture containing 25% CSA cement exhibited the most favorable balance between hydration kinetics, fresh-state behavior and strength development, indicating its potential as an effective PC–CSA mixture.

## 1. Introduction

Portland cement (PC), the primary binder in concrete, is also a major contributor to global carbon emissions, reaching approximately 1.61 billion tons in 2022 [1] and accounts for 5–9% of total global CO_2_ emissions [2,3,4]. In response to current environmental challenges, the concrete construction industry faces increasing pressure to reduce its CO_2_-related emissions by finding more sustainable alternatives. Nevertheless, portland cement remains the most widely available construction material worldwide, considering its raw components are almost globally accessible, and clinker production is projected to increase to 3180 Mt/yr by 2050 [2]. The CO_2_-related emissions from portland cement production come from both fuel combustion and from the calcination process (40% and 60%, respectively). Among the ways to address these high carbon emissions, the usage of supplementary cementitious materials (SCMs) as a partial replacement of portland cement clinker, and the usage of alternative fuels in the cement production process are the most common [5].

An alternative approach involves the utilization of non-portland clinker type of cements. One example is calcium sulfoaluminate cement (CSA), which can totally or partially replace portland cement and lower CO_2_-related emissions [5,6]. CSA cement is a family of hydraulic binders based on ye’elimite ($C_{4}A_{3}\bar{S}$) as its main component and can be produced using similar clinkering technology as for PC [7,8]. CSA cement is typically manufactured from limestone as a source of calcium, bauxite or aluminous clay as an alumina source, and gypsum or anhydrate as a calcium sulphate source [8,9,10,11]. The environmental benefits of CSA cement, in comparison to PC, are related the lower calcination temperature required for clinkering, 1250–1300 °C instead of 1450 °C, lower energy consumption in the grinding process, and reduced limestone content [5,9,10,12,13,14]. Burris et al. 2018 [14] showed that paste made with CSA cement with varying retarder dosages developed more than 80% of the 28-day strength by 3 days while PC-based paste had only reached 39% by 3 days of hydration. In the case of mortars, Huang et al. 2021 [15] also found that mixes with 100% CSA exhibited 8 and 6% greater strength than plain PC ones at 28 and 90 days, respectively. Despite improvements in strength development, CSA has shown expansion as a result of the continuous formation of ettringite and other hydrates [16,17], and reduced resistance to chloride ion penetration [18,19] due to higher permeability and lower porosity of the binder matrix, at least at early ages [20]. Also, there is no clear consensus about its shrinkage performance when compared to PC as some authors observed an increase in shrinkage for CSA-based concrete [21], while others a decrease in this effect on concrete and mortar [22,23].

Research has focused on the use of CSA together with PC as main binders in cementitious mixes with findings indicating shorter setting time with CSA cement used as partial PC replacement [15,24,25,26]. Simultaneously, these CSA additions can affect workability, with mixes often requiring retarders to maintain open time and workability [15,27]. In hardened state, small additions of CSA promoted rapid early strength of PC-CSA mixes through fast ettringite formation with higher early compressive strength [15,28,29,30]. Nevertheless, in the long term, too much CSA cement has shown to reduce later strength by hindering alite hydration, but small proportions of PC could preserve the long-term strength [25,26,31,32,33]. Besides, CSA cement can reduce drying or autogenous shrinkage by acting as a shrinkage-compensating component, but excess of this cement can lead to cracking from expansive behavior [17,31]. Durability has also the potential to be improved in terms of frost resistance, sulphate and aggressive environment-related performance and with CSA additions to PC mixes though it might also be dependent on the formulation [29,34,35,36]. Although supplementary cementitious materials (SCMs) have shown to generally moderate the rapid early reaction and setting time of CSA and CSA-PC mixes and improve their workability, refine pore structure, reduce shrinkage and result in lower CO_2_/cost, they could also slow early hydration and early strength development depending on the type and if used to heavily [37,38,39,40,41,42]. The use of CSA in combination with PC has the potential to be an attractive option as this approach would benefit the cementitious mixes from the CSA derived early strength development and the PC extended setting and workability open time but, despite ongoing research, a clear knowledge gap remains [28,43]. Additionally, the hydration behavior of CSA cement appears to be highly dependent on water availability, with properties susceptible to changing moisture conditions [32,44]. Furthermore, research has been centered on portland cement mixes with CSA as a partial replacement while blended portland cement products (e.g., mixed with fly ash), common in the European market, has not been widely studied in conjunction with CSA.

While the effect of potential PC replacements and material research is usually done changing one variable at a time, this study investigates the effect of simultaneous changes of CSA cement content, as a partial replacement of PC, and of water availability in paste and mortar mixes based on statistical design of experiments. In addition, while most research of PC replacement by CSA has focused on small replacement percentages (0–40%) [24,25,26,29], this study explores the effects of higher CSA content up to 75%. This research focused on the hydration, phases development, fresh state properties, workability retention ability through continuous mixing and early strength behavior of the mixes, including 2-h strength testing. Emphasis is placed on the interaction effect between binder composition and water availability. The study seeks to identify whether a balance between the rapid early-strength development from CSA with the extended setting and workability open time from PC can be achieved through appropriate binder design.

## 2. Materials and Methods

### 2.1. Materials

**Figure 1:**
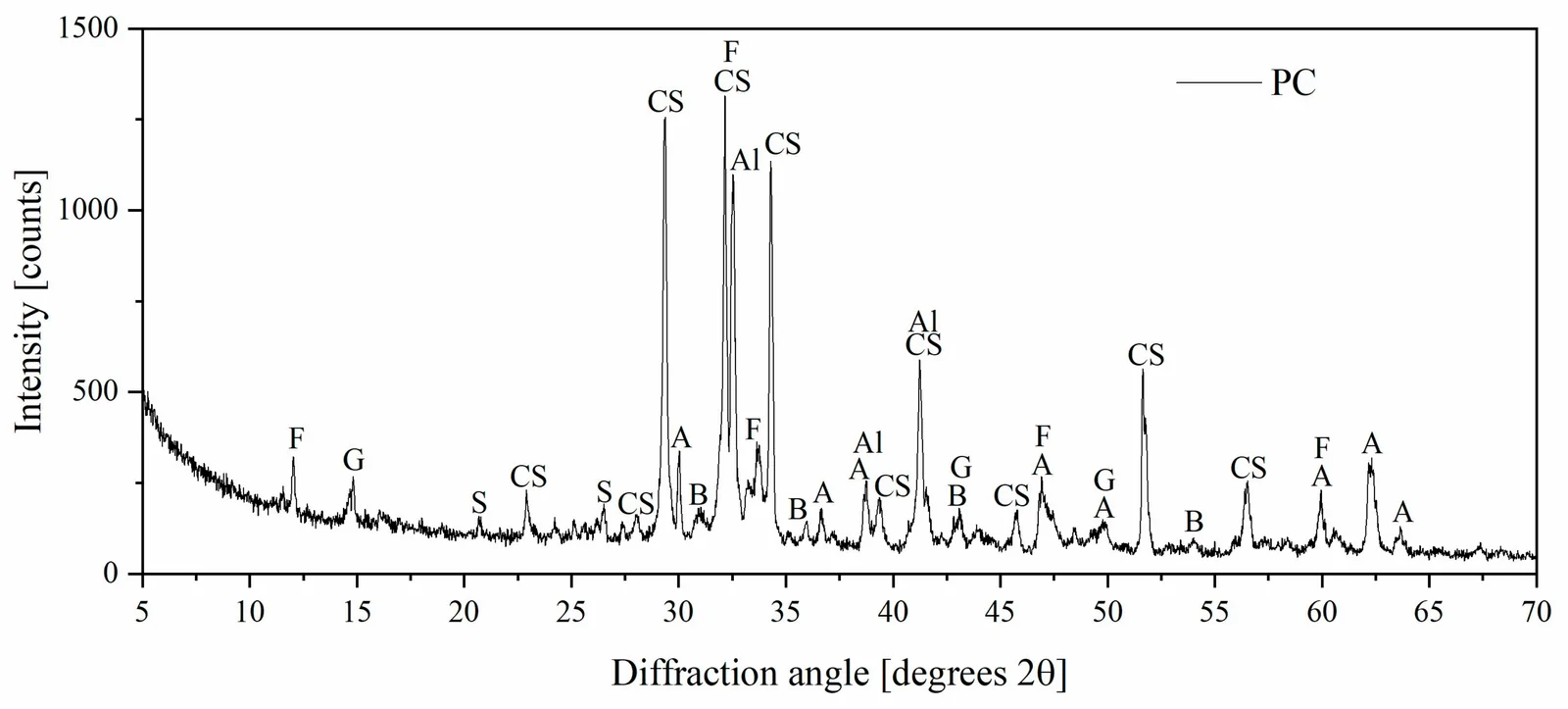
Portland cement XRD diffractogram. F—Tetracalcium aluminoferrite (C_4_AF), G—Gypsum (CaSO_4_∙2H_2_O), S—Silicon Oxide (SiO_2_), CS—Calcium Silicate phases, A—Alite (C_3_S), B—Belite (C_2_S), Al—Tri Calcium Aluminate (C_3_A).

**Figure 2:**
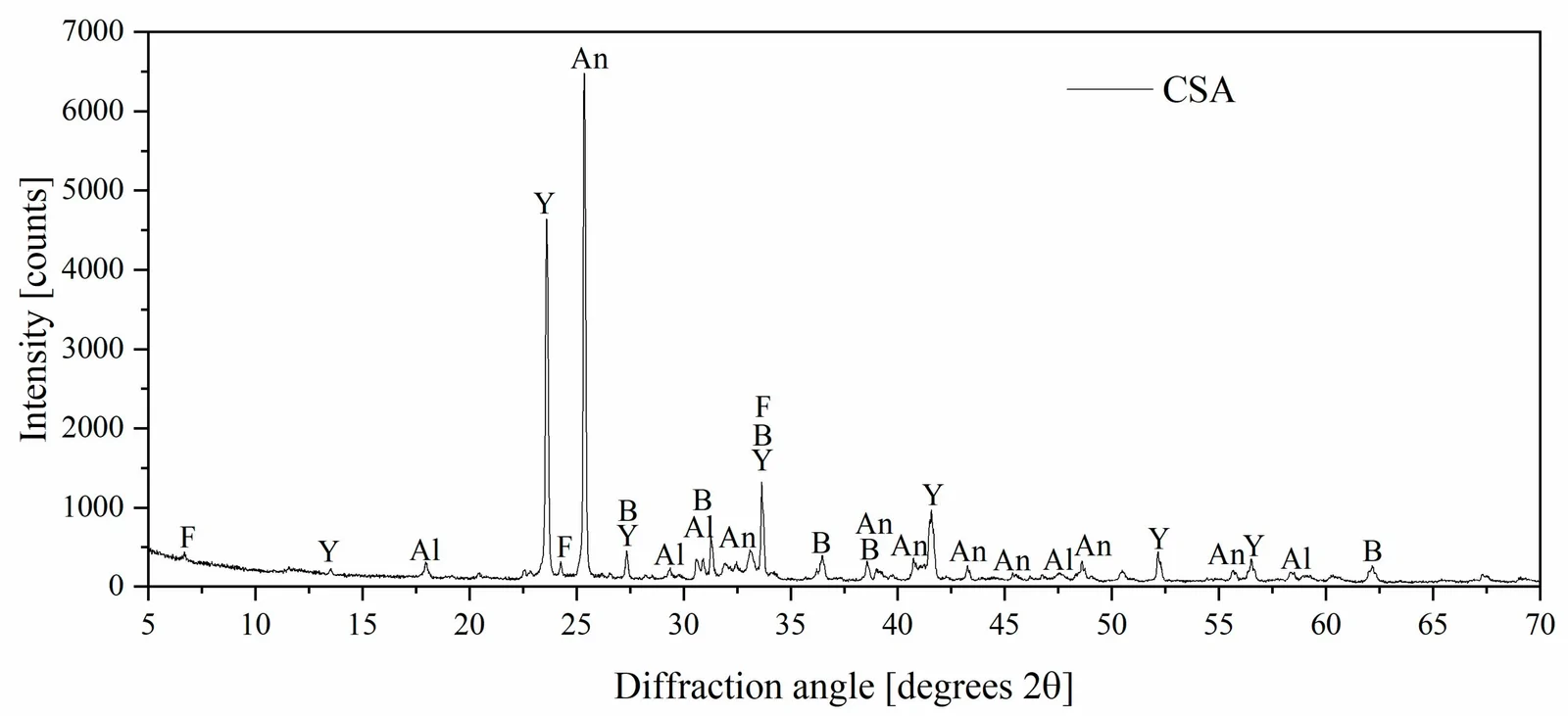
CSA cement XRD diffractogram. F—Tetracalcium aluminoferriteFerrite (C_4_AF), Y—Ye’elimite (Ca_4_Al_6_O_12_SO_3_), Al—Tri Calcium Aluminate (C_3_A), An—Anhydrite (CaSO_4_), B—Belite (C_2_S).

Two types of cement were used: PC containing fly ash (FA) and CSA. The PC, commercially available as Anläggninscement FA by Heidelberg Materials (Slite, Sweden), was CEM II 42.5 N strength class. The commercially available CSA cement was Next base SR03 by Buzzi Unicem (Trino, Italy). Clinker content of the CSA cement was 81%. Table 1 contains the main characteristics of the two cements. Table 2 contains the oxide composition of the used cements by XRF. Figure 1 and Figure 2 present the XRD diffractograms of PC and CSA cement.

The fine aggregate consisted of crushed granite sand with 0.3% water absorption and a density of 2650 kg/m^3^. MasterGlenium SKY 1966 from Master Builders (Manchester, UK) was used as a polymer-based high-range water-reducing admixture. It had a specific gravity of 1.08 and a solids content of 32 wt.%. Mixing water was tap water. No additional source of calcium sulphate was used.

### 2.2. Experimental Statistical Design and Mixture Proportions

**Figure 3:**
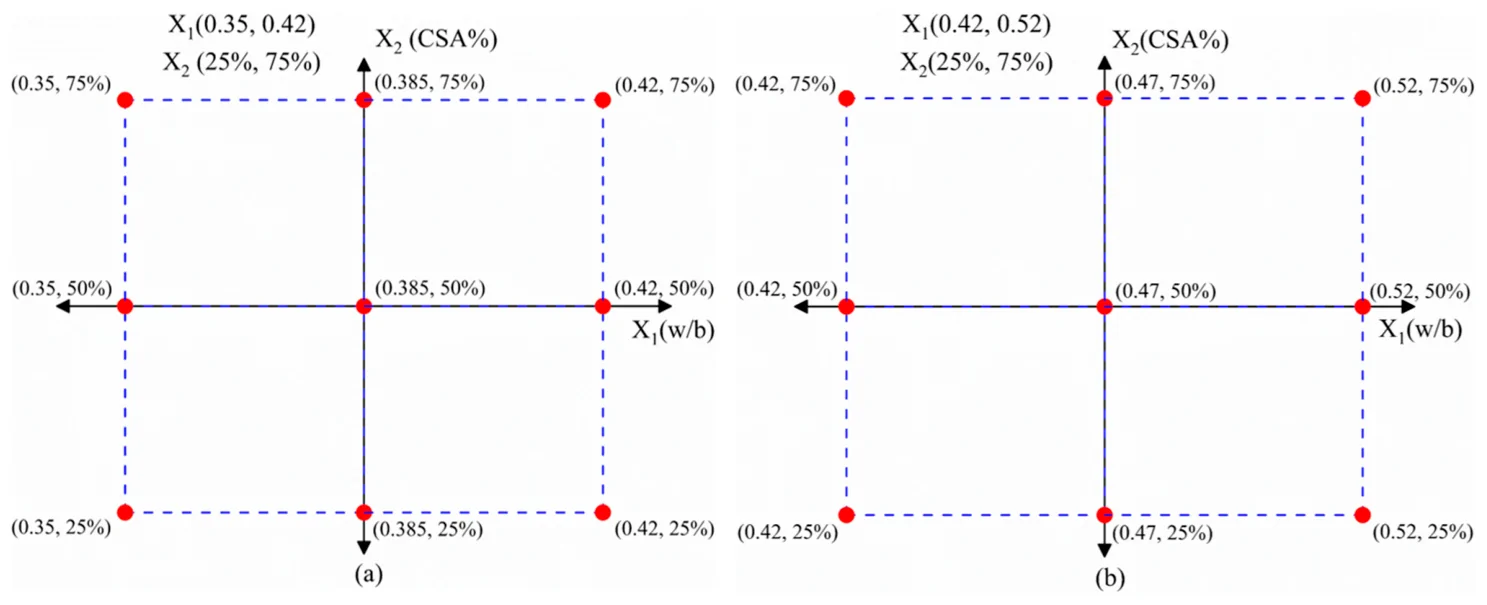
Representation of the statistical designs used. (**a**) Paste. (**b**) Mortar.

The experimental program was developed using two statistical designs to address the different test methods and specimen types. A full factorial 3^2^ statistical design with 2 replicates was used for cement paste, and a face-centered central composite design with 5 replicates on the central point was used for the mortars. Two sets of mix proportions were prepared: cement pastes for setting time measurements and mortars for flow table tests and mechanical strength. The factors studied were the amount of CSA cement as PC replacement (by mass), named CSA%, and the water/binder (w/b) ratio. Because paste and mortar present different fresh state requirements, the PC-CSA amounts are equivalent from paste to mortar, but the w/b ratio differs between them. The CSA replacements levels were 25 wt.%, 50 wt.% and 75 wt.%, while the w/b ratios were 0.35, 0.385 and 0.42 for pastes, and 0.42, 0.47 and 0.52 for mortars. Figure 3 shows a visual representation of the design matrix for pastes and mortars. The nomenclature of the mixes is defined using P or M at the start, representing paste or mortar, respectively. It is then followed by a C and the CSA percentage used, and finally by a W joined by the decimal fraction of the water/binder ratio used. For example, a mortar mix with 50% of CSA and 0.42 of water/binder ratio is designated as MC50W42. The paste mixes for setting time were done using 500 g of binder. Table 3 presents the mixture proportions of the pastes used for the setting time. The superplasticizer content was 0.1 wt.% of the total binder content for all mixes. Table 4 presents the mortar mix proportions.

The statistical designs and consequent analysis of the data were performed using the Design Expert software (v.13), with a significance level, α = 0.05, for all the evaluated responses.

### 2.3. Test Methods

Setting time was determined on pastes with the Vicat needle instrument, following the standard method EN 196-3 [45]. The paste samples were mixed for two minutes in a Hobart mixer and then placed in the truncated cone. Initial setting time was defined, as the standard indicates, as when the needle failed to penetrate more than 6 ± 3 cm, with final setting time as when the needle would not leave a full mark on the surface of the paste.

The slump-flow test was done on mortar mixes following the standard method EN 1015-3 [46]. Mortar samples were prepared using a Hobart mixer. The truncated cone was filled, then 15 manual drops from the table would promote the flow of the mortar. After the initial 2 min of mixing the spread was measured in two perpendicular directions. For the slump retention time, the test was repeated after 10 min, followed by a new measurement every 10 min until the sample had no flowability left. For each testing time, a new mortar sample was taken from the continuously running mixer.

Compressive and flexural strength were determined after 2 h, 1 day, and 7 days of curing, following the standard method EN 1015-11 [47]. Testing involved casting beam samples with dimensions of 40 × 40 × 160 mm^3^. After casting, the specimens were kept in their moulds in the laboratory at approximately 20 °C and 50–60% ambient relative humidity. The exposed surfaces were covered with plastic film to limit moisture loss. All specimens were demoulded after 2 h. Specimens scheduled for later testing were then wrapped in plastic film and kept in the laboratory until testing. The samples were first tested for flexural strength with the 3-point method. After the determination of the flexural strength, each half of the beam was tested for compressive strength. The used loading rate was 10 kN/s.

Heat flow and cumulative heat were tracked using a TAM Air isothermal calorimeter (Thermometric AB, Sollentuna, Sweden). The tests were performed on paste mixes, replacing portland cement with 0, 25, 50, 75, and 100% by mass Isothermal calorimetry was performed at a fixed w/b ratio of 0.40 to compare the effect of CSA content on hydration. This ratio allowed the samples to be prepared and transferred to the calorimeter without using a retarding admixture, which could alter the measured hydration kinetics. The sample mass was 4 g, and water was used as reference material. External mixing was used, and the filled ampoules were placed in the calorimeter in less than 5 min after mixing. The normalization of the data was performed per gram of binder.

X-Ray diffraction was performed on paste samples after 7 days of curing. Tested samples were grounded manually into powder using an agate mortar and passed through a 63 μm mesh. Zincite (10%) (ZnO; Sigma-Aldrich no. 255750, 99.99% trace metals basis, CAS 1314-13-2, Saint Louis, MO, USA) was weighed into before the final grinding step as the internal standard [48]. The powders were then loaded into single-crystal silicon holders and pressed lightly against the surface to limit preferred orientation. The test was done using a PANalytical Empyrean diffractometer in Bragg–Brentano θ–θ geometry with Cu Kα radiation (λ_1_ = 1.54060 Å, λ_2_ = 1.54439 Å) and a PIXcel3D detector (Malvern Panalytical, Malvern, UK), operated at 45 kV and 40 mA. Patterns were recorded from 5 to 70 °2θ in steps of 0.0131 °2θ.

Phases were identified by search-match using ICDD PDF-2 database (Release 2022). Quantitative phase analysis used the Rietveld method after Hill and Howard (1987) [49], run in HighScore Plus v5.1 with crystal-structure models from the Inorganic Crystal Structure Database (FIZ Karlsruhe) and the Crystallography Open Database, monoclinic (M3) alite (ICSD 94742), β-belite (ICSD 79550), cubic ye’elimite (ICSD 9560), ettringite (ICSD 155395; ref. [50]), calcium hemicarboaluminate (COD 2105252; ref. [51]), portlandite (COD 1001768), calcite (ICSD 169926), gypsum (COD 5000039), α-quartz (ICSD 89279) and zincite (ICSD 26170).

The refinements used the Chebyshev polynomial background, specimen displacement, pseudo-Voigt peak-shape coefficients, unit-cell parameters and the scale factors of the individual phases. Refinements were accepted when Rwp ≤ 12% and GoF ≤ 1.5, in line with accuracy benchmarks for multiphase hydrated cement pastes [52].

FTIR was performed in powdered samples after 7 days of curing using a thermoscientific FTIR Spectrometer (Thermo Fisher Scientific, Madison, WI, USA), removing the background with scans from 400 cm^−1^ to 4000 cm^−1^ at a resolution of 4 cm^−1^ by averaging 30 scans with the usage of the OMNIC Paradigm software (v.2.9) in % Transmittance mode.

SEM microstructural characterization was carried out using an accelerating voltage of 15.0 kV. Imaging was performed with a ×550 magnification under low-vacuum conditions and the images were obtained in backscattered-electron compositional (BEC) mode. The test was performed on polished sections from fractured mortar samples. The specimen preparation involved hydration stoppage after 7 days, followed by resin impregnation under vacuum conditions and surface polishing. In conjunction, energy-dispersive spectrometry (EDS) was performed on the same using a BRUKER energy-dispersive spectrometer (JEOL Nordic AB, Sollentuna, Sweden), connected to the SEM apparatus, and the ESPRIT software (v.2.1). EDS spot analysis was performed using the same magnification and the points were chosen based on their grey-level and apparent morphology.

## 3. Results and Discussion

### 3.1. Statistical Analysis

The results from initial and final setting time, 2- and 10-min slump, and compressive and flexural strength at different ages tests were subjected to statistical analysis based on the respective factorial design used. Table 5 presents a summary of the statistical analysis of variance ANOVA of said responses. The table shows the significant variables for each response, accompanied by a + or – sign, which points to an increasing or decreasing effect of the variable on the response, respectively, the adjusted R^2^, and the Lack-of-Fit (LoF). The analysis was performed using a significance level α = 0.05 and non-significant terms appear denoted as NS.

From Table 5 it is visible that the linear effect of the factors was statistically significant on the fresh state responses and was negative and positive for CSA% and w/b ratio, respectively. This implies that decreasing the CSA% increases the setting time and slump, while lowering the w/b ratio shortens the setting time and reduces the slump. The interaction effect between the factors was significant for both setting times and the 10 min slump. Additionally, the quadratic term of CSA% was statistically significant in the final setting time response, which suggests a non-linear response of the setting time to changes in the CSA cement amount.

For the strength responses, the linear effects of CSA% and w/b ratio were positive and negative, respectively. This indicates that, in general, higher CSA% results in improved strength and higher w/b ratio decreases it. Nevertheless, the linear effect of w/b ratio was not statistically significant on the 1- and 7-day compressive strength, suggesting that compressive strength changes at these curing ages were mainly driven by the variations in the CSA cement content. For the 1-day compressive strength response, the quadratic terms of both variables were significant and negative, suggesting a curvature in the compressive strength response surface and a lack of linearity in the effect of the studied variables at this time. The linear effect of CSA% was not statistically significant for the 7-day flexural strength, indicating that there were no governing trends because of changes in this variable in the response. The statistical analysis also showed that there was negligible interaction between the tested variables on the strength responses. Most models had relatively high adjusted R^2^ values (0.8414–0.9892). The values for 7-day compressive and flexural strength were lower, at 0.517 and 0.390, respectively, indicating limited predictive reliability. These two models should therefore be interpreted cautiously. Variability not captured by CSA content and w/b ratio may have contributed to the lower values. Possible contributors include shrinkage-related microcracking [53], isolated large pore development with increased w/b ratio and spatial distribution of the hydration products linked to incomplete hydration [54]. These properties are further explored in Section 3.6. Early-age strength. LoF remained non-significant for all the tested parameters, indicating the analysis remains useful inside the experimental domain.

### 3.2. Setting Time

**Figure 4:**
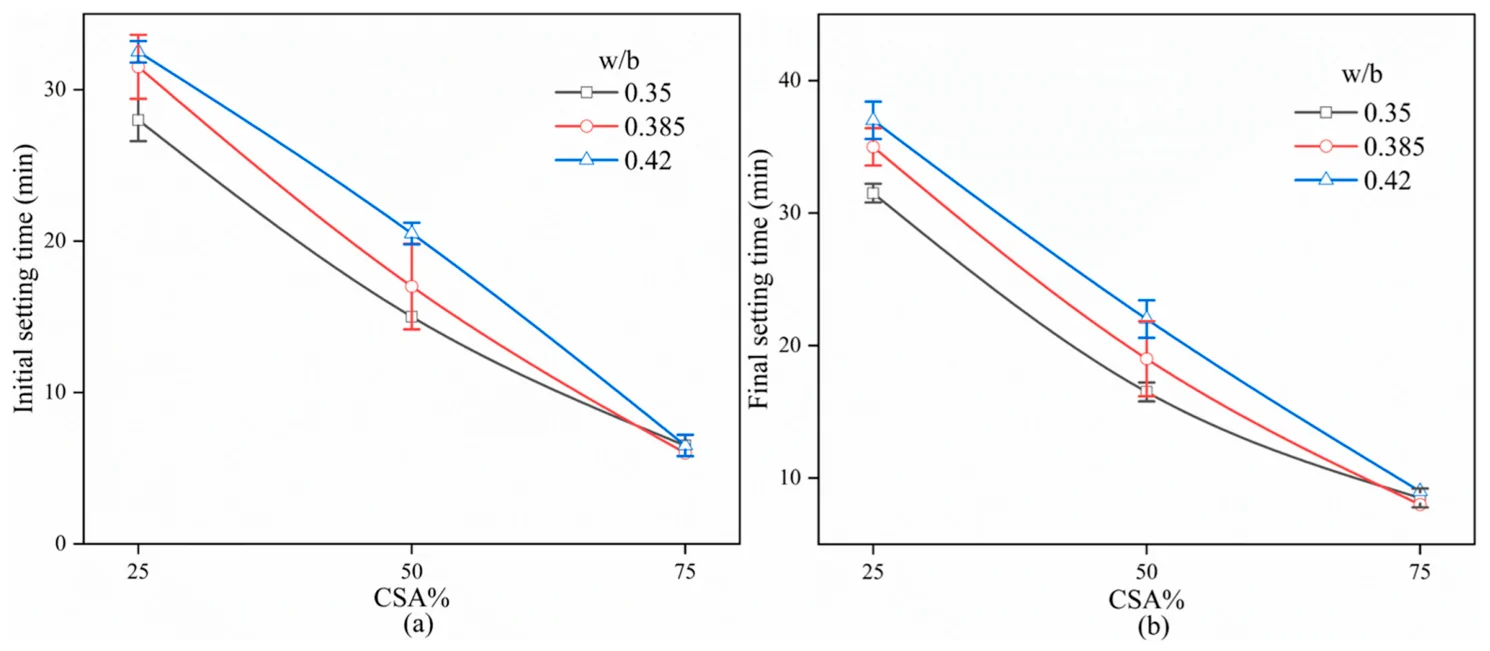
Interaction plots for (**a**) initial setting time and (**b**) final setting time.

The results from the setting time test are presented in Figure 4. The initial setting time ranged from 6.0 to 32.5 min, and the final setting time ranged from 8.0 to 37.0 min. The difference between final and initial setting time was the highest for mixes with 25 wt.% of CSA and decreased in magnitude when medium and high CSA replacement percentages were used. The largest difference between both times was only 4.5 min, registered for mix PC25W42 with 25 wt.% of CSA and 0.42 of w/b ratio. Moreover, all mixes with 25 wt.% CSA showed final setting times of more than 30 min, and all mixes with 75 wt.% CSA produced final setting times of less than 9 min. From the statistical analysis of the setting times results, visible in Table 5, the linear effect of both factors was statistically significant on the two responses. This indicates that higher CSA content resulted in shorter setting times. While the higher w/b ratio resulted in longer setting times, the statistical analysis also shows that the interaction term between CSA% and w/b ratio was significant for both initial and final setting time, which can be appreciated in the interaction plot in Figure 4. There, at 25% and 50% of CSA, higher w/b ratio increased initial and final setting times however, at 75 wt.% of CSA, the w/b ratio had no effect.

In general, the setting time of cementitious mixes is shortened when there is less water available due to the reaction of ye’elimite with calcium sulphate, rapid nucleation and dense ettringite networks, which results in rapid stiffening and faster structure formation [25,55]. Tang et al. 2015 [56] and Burris et al. 2022 [57] found shorter setting time as water to cement ratio decreased in pure CSA cement pastes. Reductions in the setting time due to the presence of calcium sulfoaluminate cement comes from the rapid reaction of ye’elimite particles with anhydrite and water [17], which results in a fast dissolved ettringite-filled microstructure [12,56,58]. These ettringite forming reactions lead to loss of plasticity, due to available water consumption, and to the rapid stiffening of the paste from the densely filled ettringite structure [59]. Ji et al. 2023 [25] also reported reductions in the initial and final setting time with increasing amounts of the CSA added to hybrid mixes with mixes of up to 8% CSA of the binder content. Qin et al. 2018 [29] presented similar findings of reduced setting time with increasing CSA content up to 20% in PC-CSA mixes.

### 3.3. Hydration Kinetics

**Figure 5:**
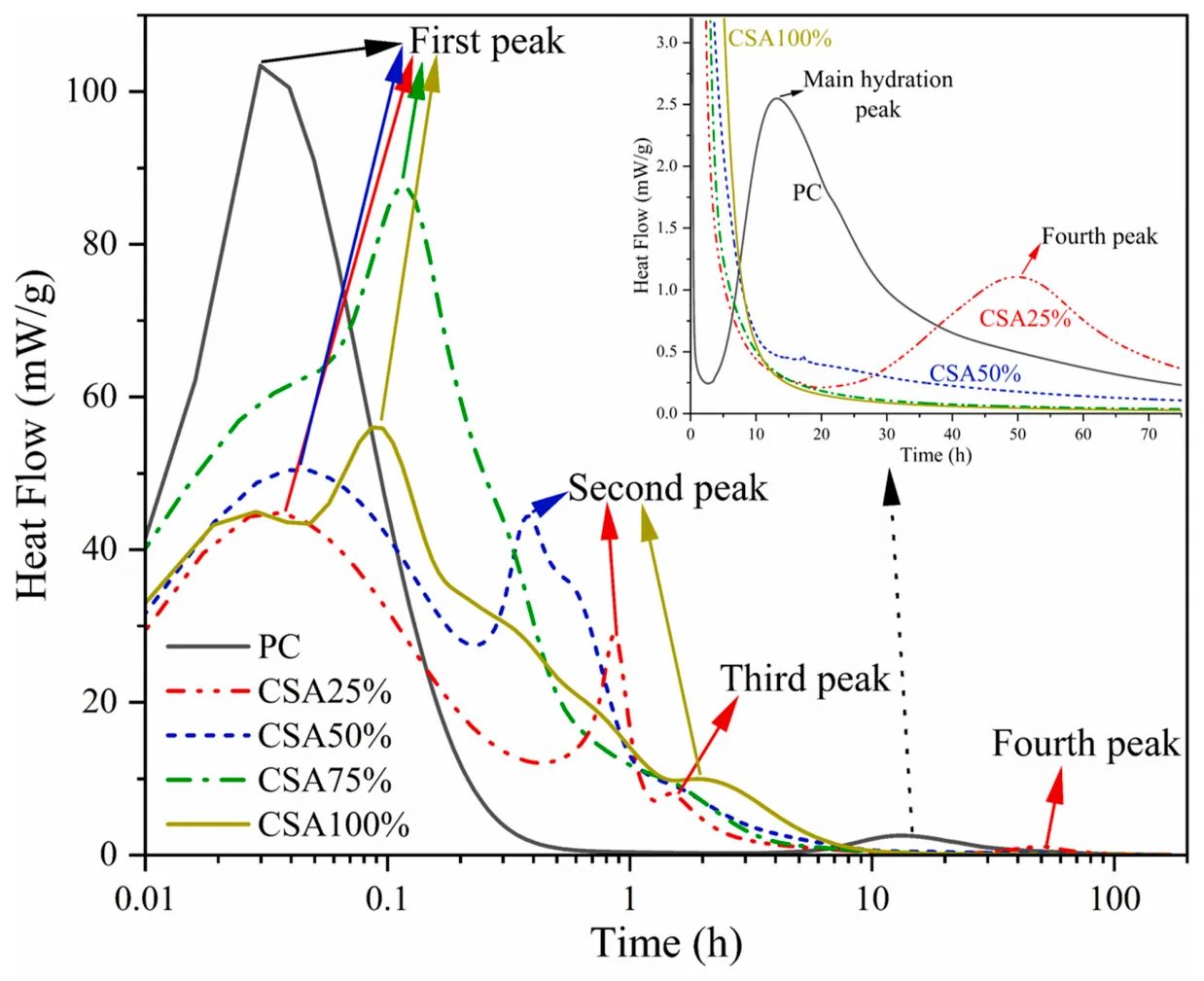
Heat flow behavior of the mixes.

The heat flow up to 7 days of the investigated cement pastes are presented in Figure 5. The results are shown as a function of hydration time for the mixture series with varying CSA percentage at a constant water to binder ratio of 0.4. The reference mix containing 100 wt.% of PC showed two exothermic peaks in Figure 5, marking the initial dissolution stage, which was followed by a prolonged induction period of about 2.7 h, and the main hydration peak at the 13.3 h mark. This behavior might be due to a retardation and smoothing effect of the main hydration occasioned by the fly ash present in the blended portland cement as well as the superplasticizer used in all the mixes. Fly ash has been reported to act as a filler material which slows down the precipitation of hydration products, extending the induction period [60]. In addition, H_2_SiO_4_^2−^, released by the fly ash, might also react with available free lime, resulting in more hydrates being produced and heat released, extending the induction period of PC [61]. CSA cement hydration is typically characterized by three exothermic peaks. The first relates to the dissolution of CSA particles. The second marks initial hydration of C_4_A_3_S and the formation of ettringite and aluminate hydroxide. The third exothermic reaction marks initiation of the hydration of the remaining $C_{4}A_{3}\bar{S}$ [62]. The third peak usually occurs at around 20–24 h after mixing with water. The principal hydration reaction of CSA cement during the first hours involves ye’elimite ($C_{4}A_{3}\bar{S}$) reacting with calcium sulphate and water to form ettringite according to:

(1) $$C_{4}A_{3}\bar{S}+2CS{\bar{H}}_{2}+34H\to C_{6}A{\bar{S}}_{3}H_{32}+2AH_{3},$$

where $CS{\bar{H}}_{2}$ represents gypsum, $C_{6}A{\bar{S}}_{3}H_{32}$ ettringite, and $AH_{3}$ aluminum hydroxide. The high heat release observed during the first 24 h for mixes with high CSA contents is consistent with the rapid kinetics of this reaction. The reaction rate is controlled by dissolution of ye’elimite and sulphate availability, followed by nucleation and precipitation of ettringite [12]. The 100% CSA mix in the present study showed the formation of the first dissolution peak at 6 min, the second after 2 h, but a third peak was not visible. After the secondary peak, a deceleration of heat release was observed, indicating possible sulphate depletion and the slowdown of ettringite formation [6,28,63]. The addition of PC to the CSA systems resulted in significant alterations in the formation of the exothermic peaks. For all tested pastes the initial dissolution, marked by the first exothermic peak, occurred within minutes after initial contact of cement with water (Figure 5). The highest peak was observed for the PC mix while, for the remaining mixes, decreasing magnitudes of dissolution peaks generally occurred in samples with increased PC content. The exception to this was for the 75% and 100% CSA mixtures, where the dissolution peak in the 75% CSA sample occurred before that of the 100% CSA sample. From Equation (1) can be seen that the presence of additional gypsums from the PC, can accelerate ye’elimite reactions [30,63,64]. The first dissolution peak, in PC-CSA mixes, is related to the reaction of C_3_A from portland cement and ye’elimite from CSA cement, which results in the initial ettringite precipitation. It is unclear why the dissolution peak for 100 wt.% of CSA appeared lower than that of 75 wt.% of CSA.

The mix containing 75 wt.% of CSA exhibited the initial heat evolution peak right after the start of the test, but no second peak was visible. This mix also showed the highest peak value from the mixes with CSA cement. It is hypothesized that the second peak was not visible due to its occurrence being accelerated and close in time to the first one as effect higher reactivity of the C_4_A_3_S with the gypsum coming from the PC, as was suggested before. Mixes with higher PC content, 25 and 50% CSA, showed peaks typical of CSA cement, including an initial dissolution peak followed by an “induction” period and formation of a subsequent secondary hydration peak plus a delayed hydration third peak which, in this case, is hypothesized to be associated with the hydration of calcium aluminate (C_3_A) from the PC [24,65]. The time to the occurrence of the second hydration peak was elongated with decreasing CSA content, from 50 wt.% to 25 wt.%. The presence of the secondary peak is likely induced by the ye’elimite reaction with calcium sulphate from the PC, which promoted ye’elimite hydration and accelerated the occurrence of the peak in relation to 100 wt.% CSA sample, but with higher heat flow [26,58,66]. Unlike the other mixtures, the 25 wt.% CSA mix exhibited a third and fourth peak. Since the third peak occurred at around 90 min, as visible in Figure 5, it is likely caused by the hydration of calcium aluminate phases coming from the PC [24,65]. The fourth peak, visible in the highlighted section of Figure 5, which interlaps with the main hydration peak of the portland cement, may have occurred due to the late hydration of alite and belite, phases that had a higher content in this mix. Ye’elimite hydration has been found to delay the hydration of calcium silicate phases in blended PC-CSA mixes [12,67,68]. The delayed peaks observed in mixtures containing higher PC content can be related to the hydration of alite ($C_{3}S$), belite ($C_{2}S$), and aluminate ($C_{3}A$) phases. Hydration of silicate phases produces calcium-silicate-hydrate (C-S-H) and calcium hydroxide according to:

(2) $$2C_{3}S+6H\to C_{3}S_{2}H_{3}+3CH$$

(3) $$2C_{2}S+4H\to C_{3}S_{2}H_{3}+CH$$

**Figure 6:**
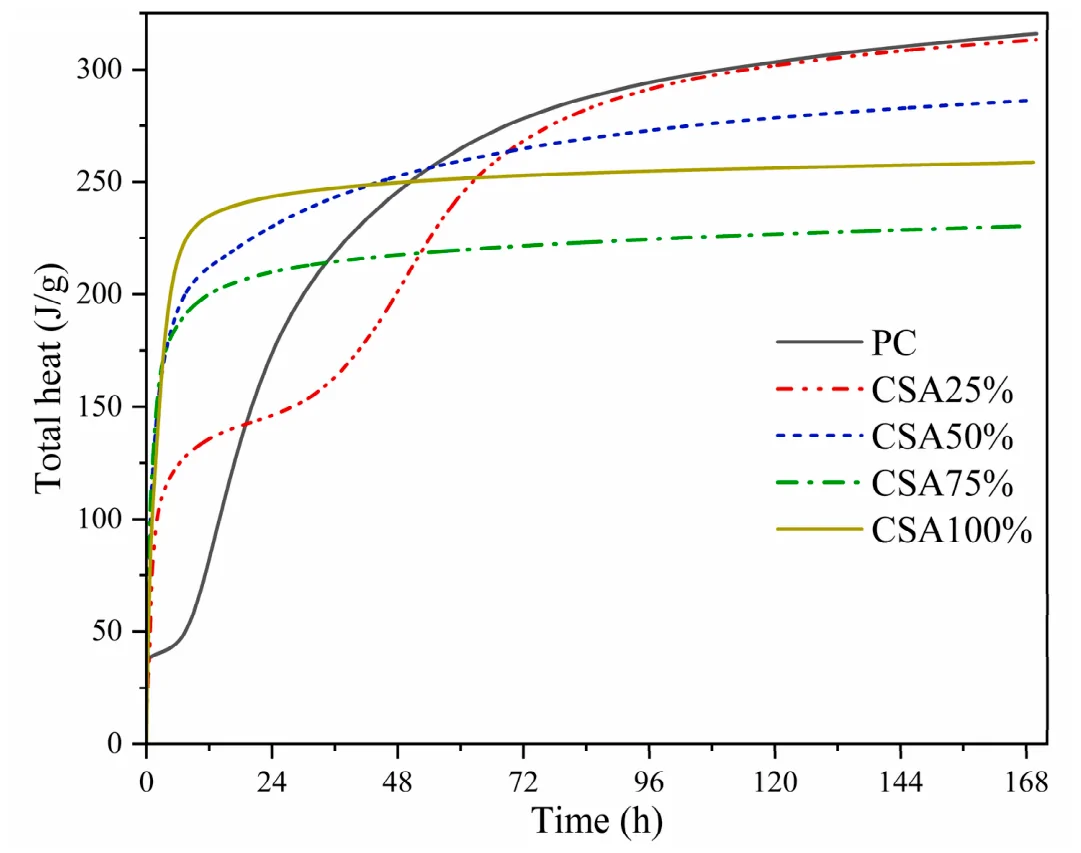
Cumulative heat of the mixes up to 168 h.

These reactions proceed more slowly than ye’elimite hydration and continue for several days. Figure 6 presents the total heat released by the mixes up to 7 days of curing. The highest increase in cumulative heat by CSA-containing mixtures appeared during the first 24 h of hydration because of the rapid hydration of ye’elimite and consequent ettringite formation, similar to the results shown by Huang et al. 2019 [69]. At later times, mixes containing 50–100 wt.% CSA showed lower heat release in comparison to mixes containing 25 wt.% CSA and the PC mix. Mixes with higher PC content continued to show higher accumulated heat release after the acceleration stage, which, from a kinetic viewpoint, indicates that hydration rates remained active for longer periods due to continuous hydration of alite and belite, Equations (2) and (3), while high CSA% mixtures experienced rapid early reaction rates controlled by ettringite formation but with a faster rate reduction after sulphate consumption and reactive surfaces coverage by hydration products. Similar observations were suggested by others [29,58,70]. The continuous hydration of PC suggests that the mixes would continue to develop more strength with time, while mixes consisting of higher CSA content would reach a high portion of their ultimate strength during the first hours after mixing with water, as similar studies have found before [15,33]. Table 6 presents a summary of the heat of hydration descriptors: the duration of the induction period and the time to the main hydration peak, measured from the start of the test and the total heat values measured after 24 h (Q_24_), 72 h (Q_72_), and 168 h (Q_168_).

### 3.4. X-Ray Diffraction and Rietveld Quantification

**Figure 7:**
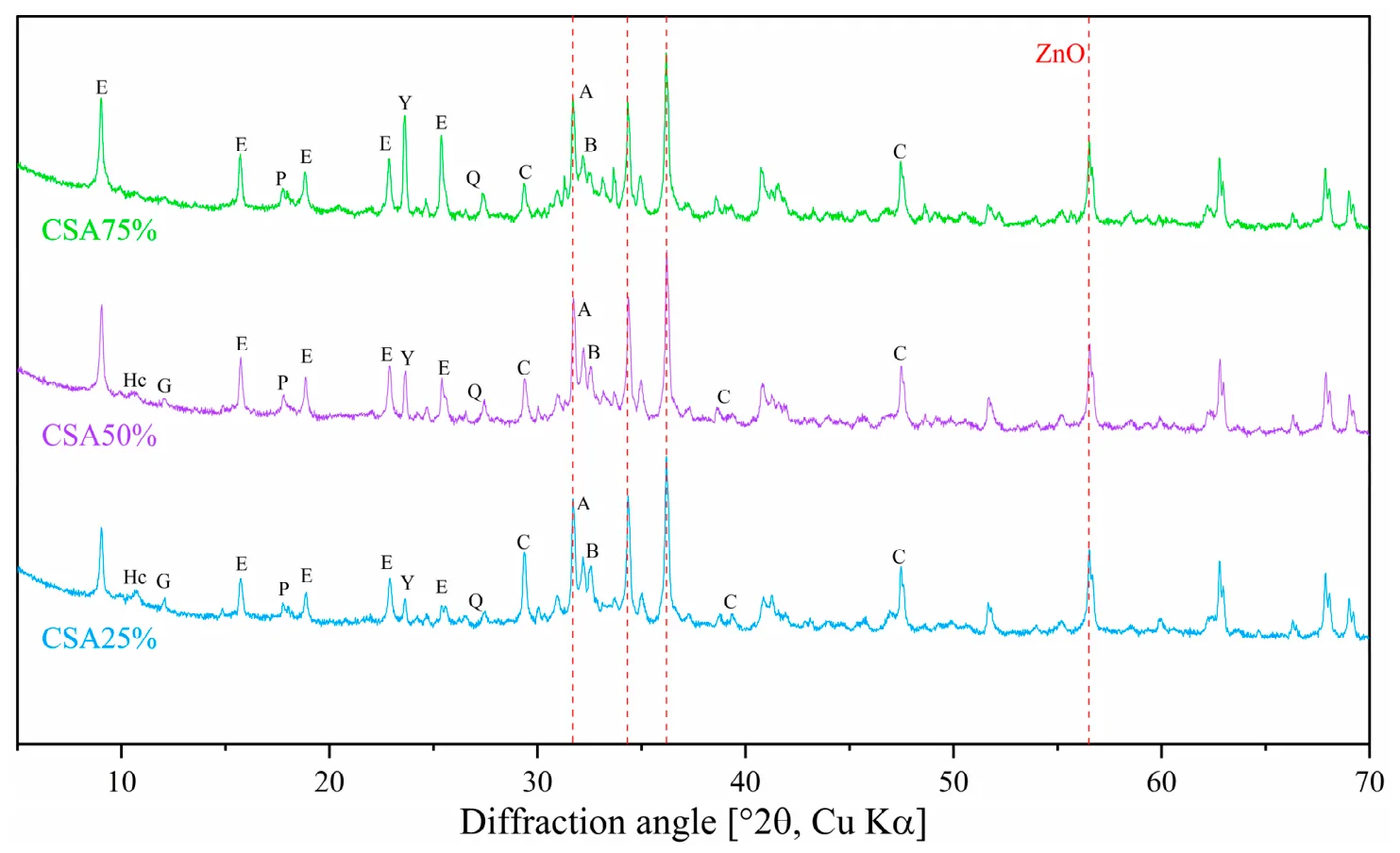
X-Ray diffraction. E—Ettringite (Ca_6_Al_2_(SO_4_)_3_(OH)_12_∙26H_2_O), P—Portlandite (Ca(OH)_2_), Hc—hemicarboaluminate (Ca_4_Al_2_(CO_3_)_0.5_(OH)_13_·5.5H_2_O), Q—Quartz (SiO_2_), C—Calcite (CaCO_3_), A—Alite (C_3_S), B—Belite (C_2_S), Y—Ye’elimite (Ca_4_(AlO_2_)_6_SO_4_), G—Gypsum (CaSO_4_∙2H_2_O); dashed red lines = ZnO internal standard.

Figure 7 shows the XRD spectra for mixes with a 0.42 w/b ratio after 7 days of curing and three CSA% replacement levels: 25%, 50%, and 75%. Table 7 presents the results from Rietveld refinement and quantification analysis.

The XRD diffractograms show ettringite, portlandite and monosulfoaluminate as the main crystalline hydration products forming from the hybrid mixes. Rietveld refinement and quantification also revealed the presence of amorphous content in all the hydrated pastes studied, attributed to C-(A)-S-H gel. Higher ettringite and ye’elimite peaks are visible as the CSA cement content increases while the quantification confirms the rise in these phases content from 25% to 75% CSA. In contrast, the quantified amorphous content decreases with higher CSA cement percentage, expected as the main hydration product from PC is amorphous C-S-H gel. Gypsum peak was not visible for 75% CSA and its content is the lowest in the 50% and 75% CSA mixes, indicating a high sulphate consumption in the reactions. Alite and belite peaks are visible in the cement pastes spectra, regardless of the CSA cement amount, which could be caused by the fast hydration of the ye’elimite, as Bolaños-Vásquez et al. 2020 [68] suggested. In a study with CSA content up to 20% in hybrid PC-CSA blends, Qin et al. 2018 [29] suggested a growth in the intensities of the alite/belite phases as the CSA percentage increased, due to CSA cement acting as a hydration inhibitor of the PC particles [71,72]. Consequently, similar behavior can be observed in the current study with higher intensity of alite/belite with increasing CSA cement content, visible in Figure 7 and confirmed by the Rietveld quantification in Table 7 with increasing content of both calcium silicate phases from 25% to 75% CSA. Furthermore, the lack of anhydrite peaks and low gypsum indicate the almost to full consumption of calcium sulphate sources and the consequent transformation of AFt into AFm, represented by the Hc, caused by excess calcium aluminate phases [29,73].

### 3.5. Fourier Transformation Infrared Spectroscopy (FTIR)

**Figure 8:**
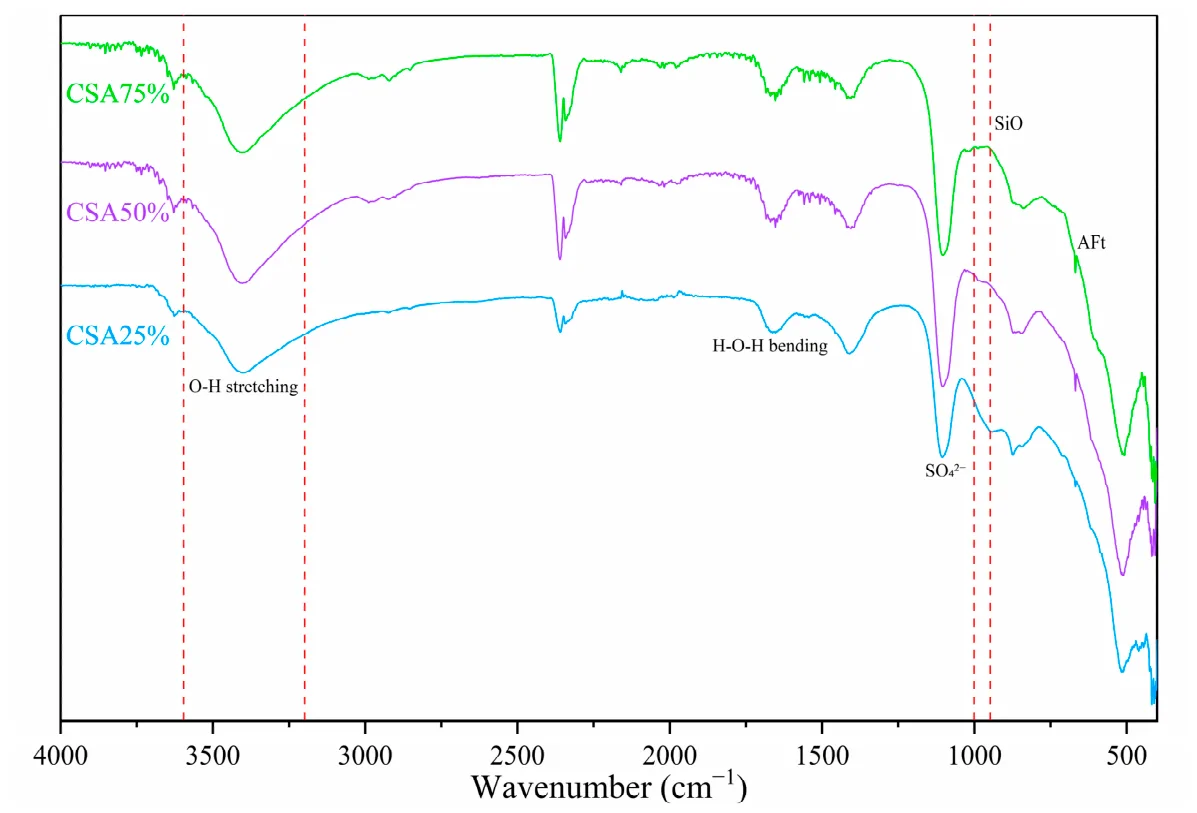
FTIR spectra of hydrated PC-CSA pastes at 7 days.

Figure 8 presents the FTIR spectra of samples with a 0.42 w/b ratio at 7 days of curing with three CSA% replacement levels: 25%, 50%, and 75%.

The FTIR spectra of the hydrated PC-CSA blends with 25%, 50% and 75% CSA in Figure 8 indicates changes in the hydration products as the CSA cement content increases. The O-H stretching band in the 3200–3600 cm^−1^ region, associated with structurally bound water in ettringite, C-(A)-S-H/C-S-H gel and other hydrated phases [74,75], becomes broader and more intense with increasing CSA content, particularly for 50% and 75% CSA, suggesting a greater concentration of hydration products containing chemically bound water. This behavior is consistent with the results from isothermal calorimetry and XRD which have shown a stronger reaction and higher ettringite content, respectively, with higher CSA cement content and agrees with ettringite precipitation from ye’elimite early hydration. The absorption band near 1100–1120 cm^−1^, which is assigned to sulfate SO_4_^2−^ vibrations in ettringite [76,77], becomes more pronounced with increases in CSA content, while the bands in the 600–700 cm^−1^ region, associated with sulfate bending modes [78], also increase. This continues to suggest a higher AFt content resulting from increasing CSA cement percentage, as XRD quantification showed. The Si-O stretching region near 950–1000 cm^−1^, which reflects the coexistence of silicate and aluminum hydrates from portland and CSA cement, respectively, exhibited changes with increasing CSA content [75,76]. These changes suggest the polymerization state of the hydrate network and confirm the evolution of the hydrated matrix from a predominantly C-S-H characterized system with 25% CSA towards a mixed AFt/C-(A)-S-H blend as 75% CSA is used.

Even though changes in the w/b ratio were not tested, it is hypothesized that an increase in w/b ratio would increase the amount of free and weakly bound water in the hydrated matrix, modifying the hydrogen-bonding environment of AFt and C-(A)-S-H/C-S-H phases. Although additional water may facilitate hydration, the resulting reduction in hydrate packing density and increase in capillary porosity would weaken nanoscale cohesion and ultimately reduce compressive and flexural strength [55,57].

### 3.6. Flow Table Slump (Mortar)

**Figure 9:**
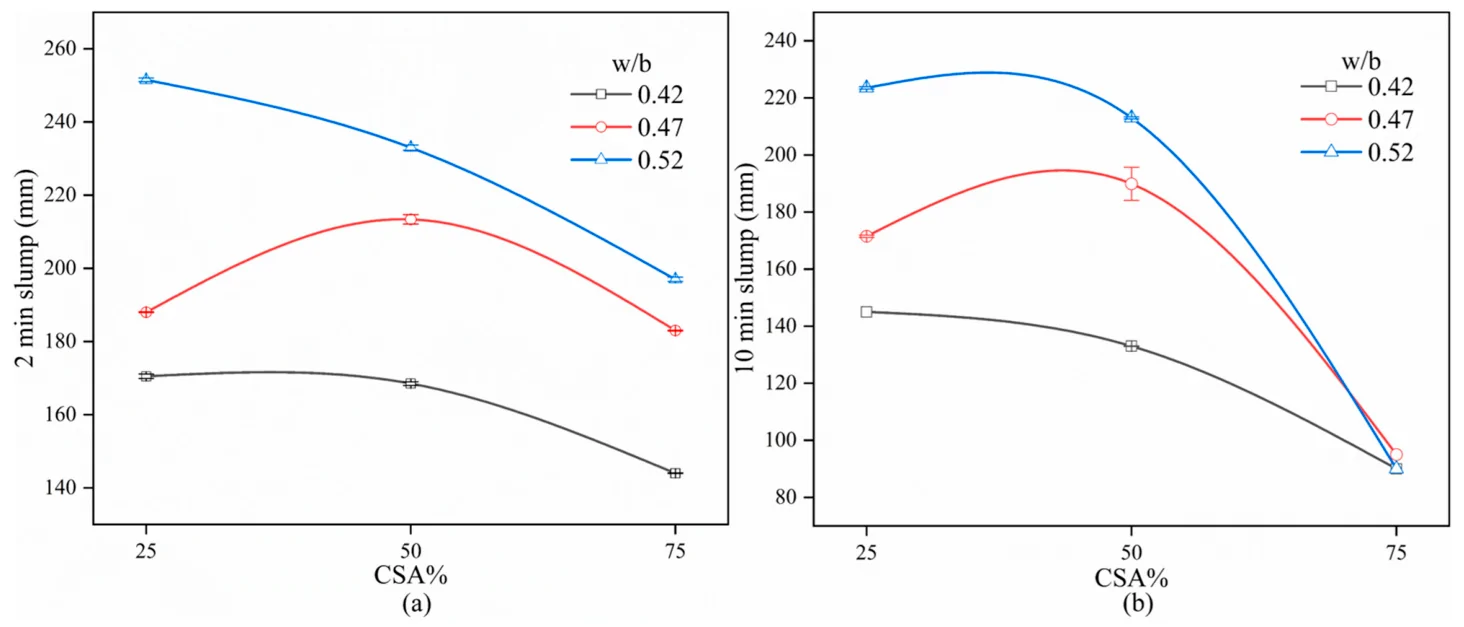
Interaction plot for slump flow of mortar samples by w/b ratio series measured after (**a**) 2 min, (**b**) 10 min.

The results obtained after 2 and 10 min of mixing are presented in Figure 9 as an interaction plot between the variables of CSA% and w/b ratio. The 2-min slump ranged from 144.0 mm (mix MC75W42) to 251.5 mm (mix MC25W52), indicating that the slump flow was the lowest for the mix with the highest amount of CSA and the lowest w/b ratio, and largest for the opposite conditions. The same behavior was observed for the 10 min measurement, but flow magnitudes were reduced and varied from 90.0 mm to 223.5 mm. Slump values of 90 mm indicate that the sample had no flowing ability and kept the shape of the slump cone.

From the statistical analysis summary, presented in Table 5, the quadratic term of CSA% was statistically significant as well on both tested times, which suggests a non-linear response of the slump to changes in the CSA cement amount. The linear effect of CSA cement was negative, indicating a decrease in slump with increasing amounts of cement in the mortar and, conversely, the w/b ratio linear effect was positive, showing slump growth with more water content. For all the CSA amounts tested, increases in the w/b ratio led to a wider slump spread, indicating that water content was a primary driver of initial workability. Considering the effect of changes in the CSA amount, for mortars with 0.42 and 0.52 of w/b ratio, the presence of more CSA resulted in decreased slump. Nevertheless, the series with a 0.47 w/b ratio showed an increase in slump when CSA changed from 25% to 50% but was followed by a decrease when CSA content reached 75%. The slump-decreasing effect from CSA and increments in its content in hybrid mixes is related to the fast hydration of ye’elimite and ettringite formation, which consequently leads to the taking up of available water, pore structure and porosity aggravation due to the accelerated formation of large ettringite structure formation [29], which also is in accordance with the registered decrease in setting time in this study. Moreover, one mole of ye’elimite requires 38 moles of water to form ettringite, which also occurs quickly, leaving less accessible free water between the blended particles [79]. For instance, Huang et al. 2021 [15] observed improvement in workability with increasing PC content added to PC-CSA blended mixes, which resulted in more available free water.

After mixing for 10 min, all mixes presented less slump flow than at 2 min. This slump loss with time, regardless of continuous mixing, might also be generated due to the initial dissolution effect of ye’elimite and aluminate phases reacting with water, which leads to the initial ettringite formation and less free water availability, as has been discussed before [29,79]. Table 4 shows that the interaction effect between the variables became statistically significant after 10 min of mixing. This suggests a time-dependent behavior of the variables to condition one another, with continued hydration promoting slump diameter reduction in time. The interaction can be understood as the effect of CSA% dominating over the influence of w/b ratio changes. Figure 9 shows this behavior with parallel lines for the w/b ratio series when CSA% went from 25% to 50%, but when CSA cement amount changed from 50% to 75%, all three w/b ratio series met around slump values of 90–95 mm regardless of the w/b ratio used.

**Figure 10:**
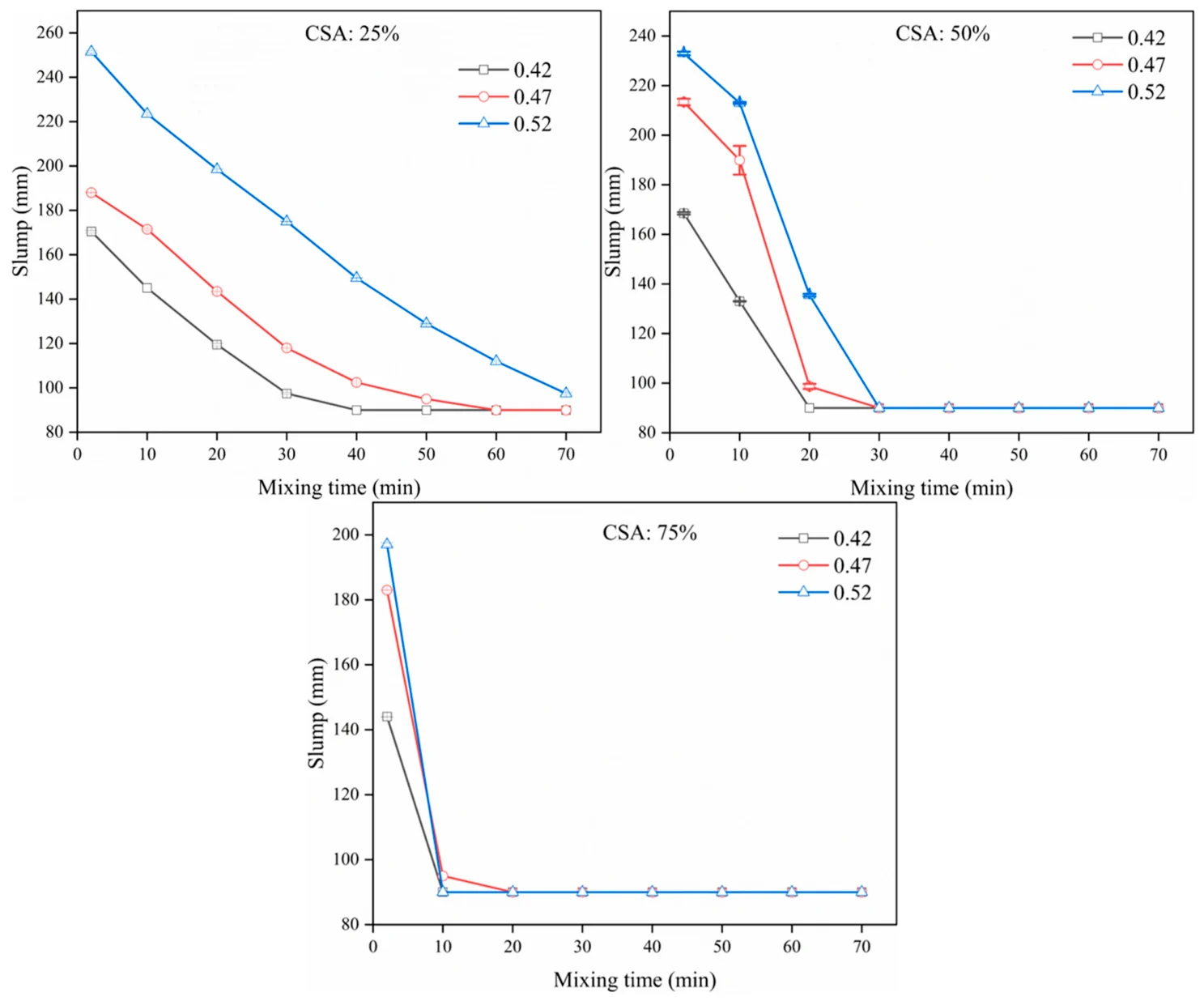
Slump retention time for all mixes.

Slump retention time continued to be tested at elapsed times every 10 min after the initial 10 min of mixing up to 70 min (Figure 10). After 10 min of mixing all mixes with 75 wt.% of CSA had no flowing ability, indicating this CSA content produced workable mixes only for tests done after 2 min. Mixes with 25% CSA were able to retain workable behavior after mixing for up to 30 min (slump ≠ 90 mm) but, regardless of increases in the w/b ratio, no mixes with 50 and 75 wt.% CSA preserved workability. Results showed that increasing the CSA cement content resulted in shorter setting times and lower slump retention ability. Moreover, additional continuous mixing did not improve the workability retention time of the mixes. Workability retention was achieved only through increased w/b ratio.

### 3.7. Early-Age Strength

Table 8 presents the mean compressive and flexural strength values of the tested mortar mixes at 2 h, 1 day, and 7 days. All the mixes exhibited sufficient strength to be tested 2 h after mixing. In general, compressive and flexural strength tended to grow with increasing CSA cement content and decreased with higher w/b ratios.

From the statistical analysis shown in Table 5, CSA content had a statistically significant positive effect at all tested ages, meaning the compressive strength is promoted by increasing the CSA content used in the hybrid mixes, which is also visible in strength values in Table 8. Conversely, the w/b ratio had a negative effect but was only statistically significant at 2 h of curing, as shown in Table 8, with compressive strength diminishing with higher w/b ratio. The appearance of the quadratic terms of both variables as significant and negative for the 1-day compressive strength, as the statistical analysis and Table 5 presented, is visible as for mixes with 25% CSA strength decreased when the w/b ratio increased; however, mortar mixes with 50 and 75% CSA showed an improvement in compressive strength when w/b ratio changed from 0.42 to 0.47. As the slump response in Figure 9 showed, this increase in w/b ratio also improved the workability of the mixes which can be linked to an improved compaction ability of the mixes with more available water, or even more efficient ye’elimite hydration and ettringite formation with an increased w/b ratio [80,81].

As the statistical analysis showed, CSA% had a positive effect on flexural strength leading to the development of more flexural strength with higher CSA content. This is particularly visible in the strength behavior at 2 h of curing but, at 1 day of curing, mixes with 0.52 w/b ratio showed a steep increase in flexural strength as CSA% changed from 25 to 50% but, 75% CSA diminished the flexural strength from the former, in line with the significant negative quadratic effect of the CSA%, A^2^, visible in Table 5, and could be derived from excessive ettringite formation which creates high crystallization stresses, leading to microcracks, and possible expansion before enough rigidity is formed [17,82]. The linear component of the w/b ratio had a significant negative effect at all tested ages, particularly visible at 2 h of curing, with consistent strength reductions with higher w/b ratio. At 1 day of curing, the effect of w/b ratio was not clear for mixes with a higher CSA%. At 50% CSA, strength increased as w/b ratio changed from 0.42 to 0.47 but dropped with 0.52 w/b ratio. For mixes with 75% CSA, 0.42 and 0.47 w/b ratio resulted in the same strength indicating that the effect of CSA is predominant over that of the w/b ratio and only 0.52 w/b ratio resulted in lower strength. The statistical analysis showed that at 7 days of curing only the w/b ratio was significant over the flexural strength. Table 8 showed this effect for the evaluated CSA cement content and this indicates that w/b ratio changes were dominant at this later age over the behavior of flexural strength. Changes in CSA% were consistently significant for compressive strength while w/b ratio had a clearer influence on flexural strength.

**Figure 11:**
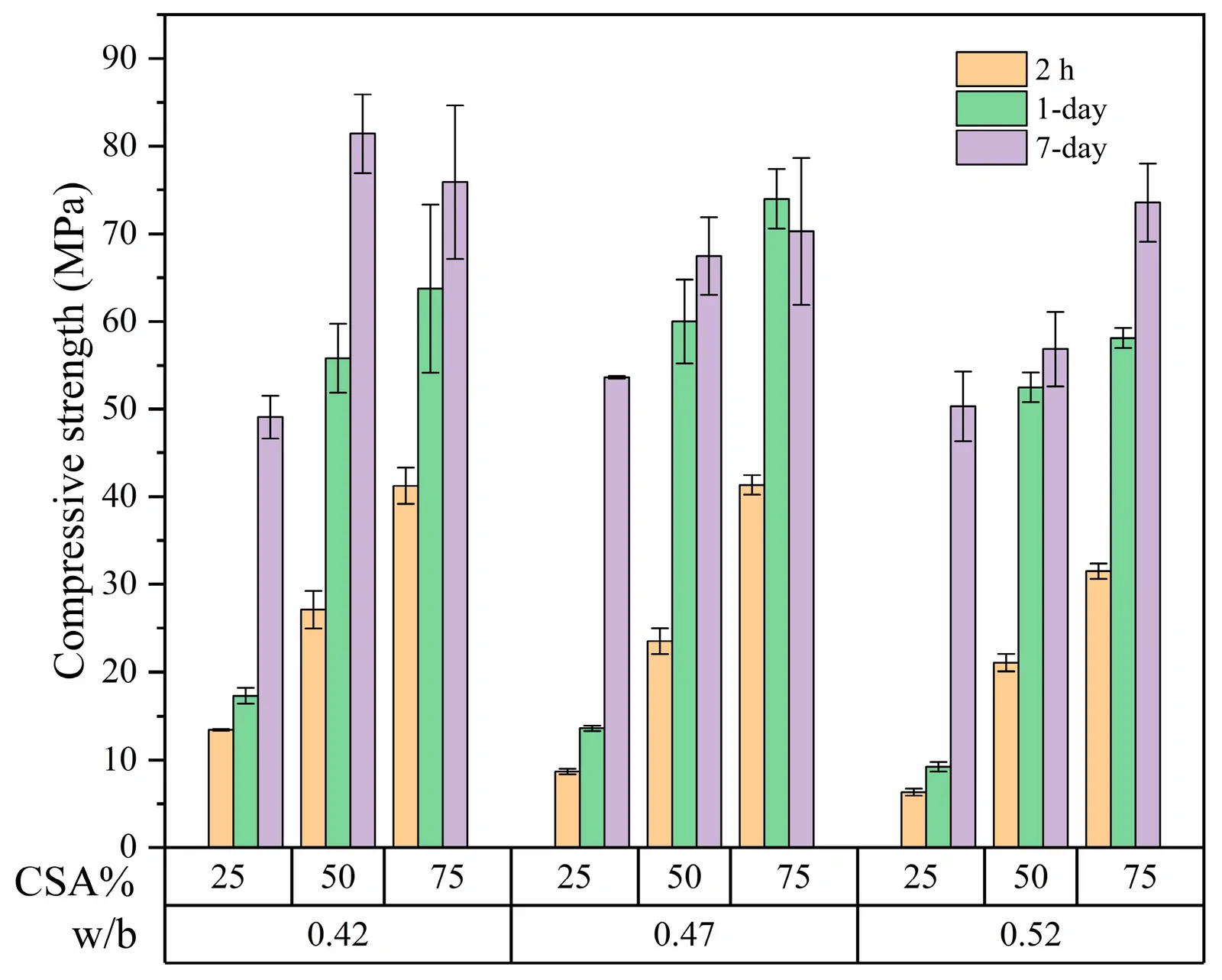
Compressive strength evolution of the mixes at 2 h, 1-day and 7-day.

Figure 11 shows the compressive strength evolution with time of the tested mortar mixes. It is possible to observe that the highest 2-h compressive strength, mix MC75W47, was not predictive of the highest 7-day strength, mix MC50W42. In addition, the lowest 2-h compressive strength, MC25W52, resulted in the comparable lowest 7-day compressive strength which were produced by the mixes with the least CSA cement content. Moreover, it can be appreciated that the strength growth from 2 h to 1 day was the lowest for mixes with less amount of CSA while the development was generally the highest for mixes with 50% and 75%. Conversely, from 1 day to 7 days, the development was more pronounced in mixes with 25% of CSA and lower for mixes with moderate to high amount of CSA cement.

The presence of CSA cement in cementitious mixes is often related to increased early compressive strength [29,30,66,83]. For instance, Ji et al. 2023 [25] reported a 29.9% increase in 3-day compressive strength when using 8% of CSA cement. Additionally, Lee at al. 2020 [84] suggested an optimal PC replacement by CSA of 17% after reaching a maximum of 8.3 MPa in compressive strength at 12 h without compromising the workability. This improvement in the compressive strength at early ages can come from the higher hydration rate of CSA cement [29]. Nevertheless, compressive strength reductions at 7 days of curing and later ages in cases of CSA replacing more than 10% PC have been reported, with authors relating these decreases to expansion induced microcracking by the growth of AFt and limited hydration of PC by CSA cement fast hydration [25,29,43,85,86]. The present study found sustained increases in strength with more CSA cement and at every w/b ratio tested. Studies in which CSA cement was used as the main binder and was partially replaced by PC showed that 10–30% PC resulted in up to 60% decrease in 1-day compressive strength [58], and 10–40% PC as CSA replacement initially increased the 2-h compressive strength but the effect weakened with time [30].

**Figure 12:**
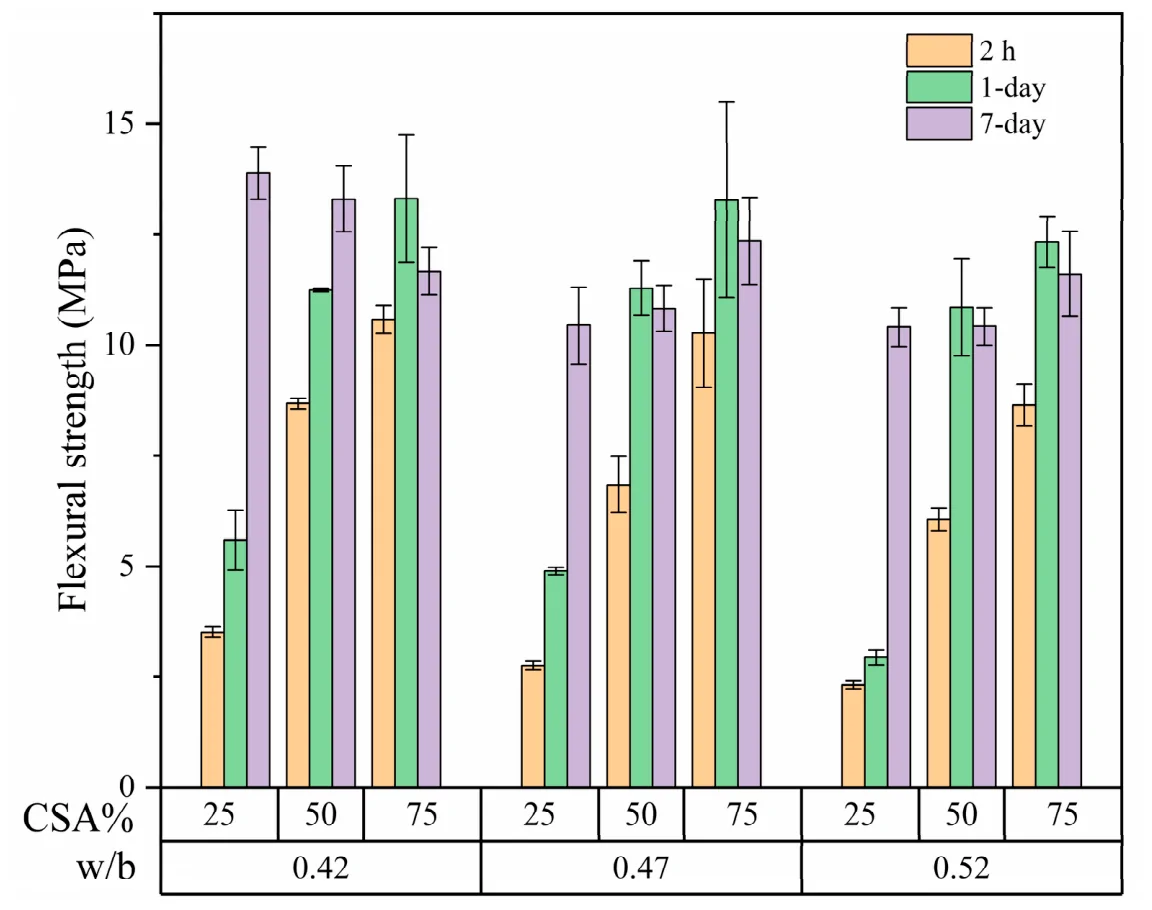
Flexural strength evolution of the mixes at 2 h, 1-day and 7-day.

Figure 12 presents the flexural strength evolution with time of the tested mortar mixes. In general, it increased from 2 h to 1 day, but the later development varied depending on the mixture composition. The most consistent strength gain was observed from 2 h to 1 day of curing, particularly for mixes with medium to high CSA contents. From 1 to 7 days of curing, strength evolution continued for mixes with 25% of CSA, but 50 to 75% of this cement showed a limited increase and, in some cases, even lower mean strength, particularly in mixes with a higher w/b ratio, but within the statistical experimental scatter. Xie et al. 2023 [31] attributed the appearance of holes and cracks to the conversion of ettringite into monosulfate which resulted in a strength decline with time. In this study, the mix with the highest 2-h flexural strength, MC25C42, was predictive of the highest 7-day flexural strength.

Flexural strength reductions with time on blended PC-CSA mixes are often associated to expansion behavior of ettringite and increased crystallization stress, which are promoted by higher CSA cement content [17,25]. Regardless, 2-h and 1-day flexural strength improved consistently with higher CSA cement content but reductions in flexural strength from 1 to 7 days, were observed when 75% CSA, for any w/b ratio, and for 50% CSA but with 0.47 and 0.52 w/b ratio.

In the current study, 25% CSA as PC replacement allowed for consistent improvement of compressive and flexural strength, regardless of the w/b ratio. 50% CSA gave the highest compressive strength and promoted flexural strength at 0.42 of w/b ratio but might drop at higher ratios. Finally, even though 75% CSA resulted in the highest 2-h compressive strength, 1-to 7-day flexural strength experienced a decline, regardless of the water content.

### 3.8. SEM Observations and EDS Analysis

**Figure 13:**
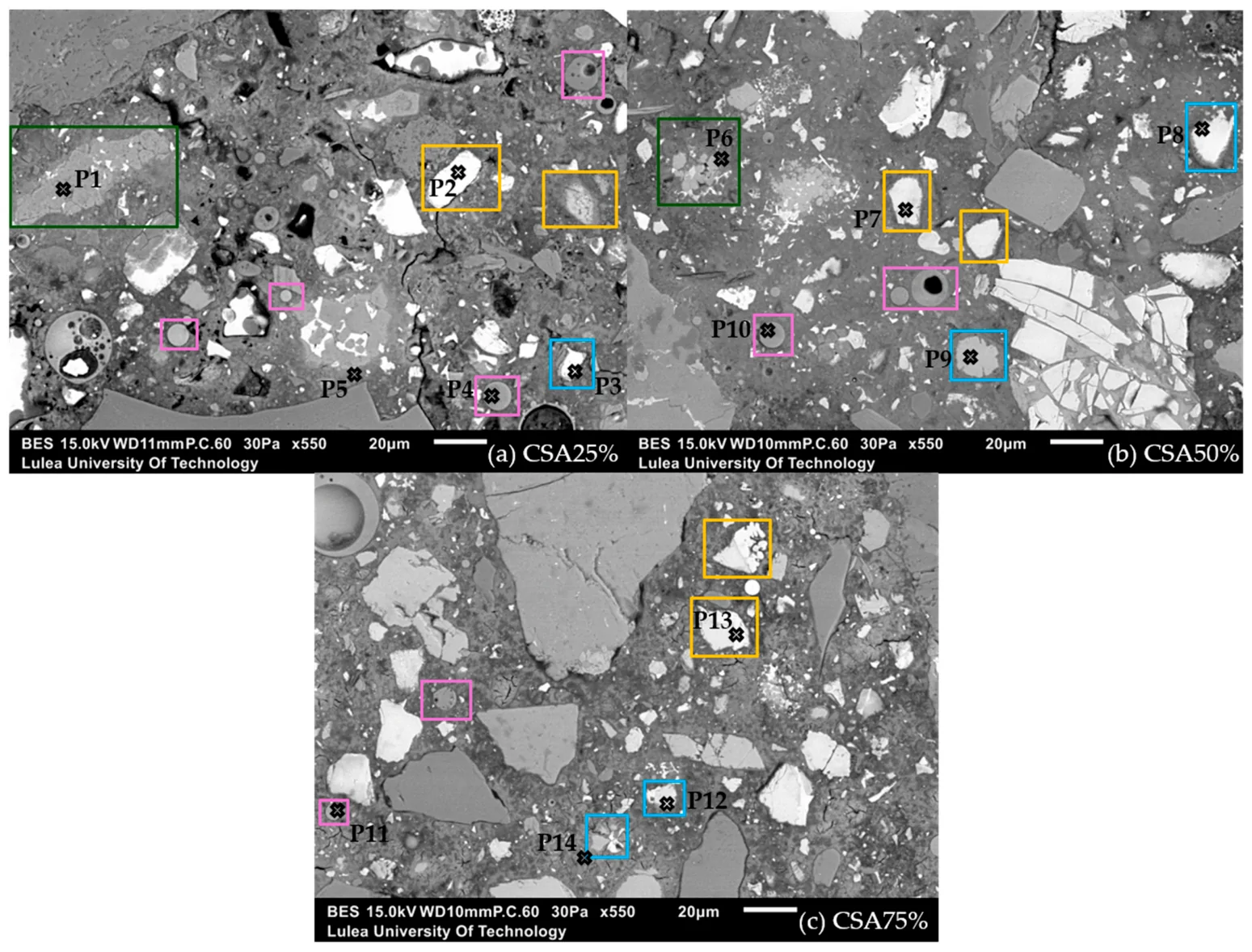
SEM images at ×550 for mortar samples at 7 days of curing with a w/b ratio of 0.47. (**a**) 25% CSA, (**b**) 50% CSA, (**c**) 75% CSA. Blue boxes indicate CSA, yellow boxes PC, magenta boxes FA and green boxes C-(A)-S-H. EDS points range from P1–P14 and the X indicates the position of each.

Figure 13 presents the SEM images for mortar samples with a w/b ratio of 0.47 and 25, 50, and 75 wt.% of CSA amount after 7 days of curing. Visual comparison of the electron microscopy images revealed changes in the amount of hydration products, unhydrated phases, and density of the internal structure of the mortars. The images show the presence of unhydrated cement particles from CSA, PC, and fly ash (FA). PC and CSA cement particles are characterized by brighter appearance than hydration products, and FA particles are distinguished by their distinctive circular shape. Darker regions were interpreted as porosity. The SEM images suggest that the amount of unreacted cement and FA decreases as the CSA percentage increases, due to mixture composition. Nevertheless, some of the PC and CSA particles remained partially hydrated and unhydrated, regardless of the CSA content, signaling the early hydration age of the mixes and that the amount of available water might have been too low for the full hydration of ye’elimite. Previous research indicated that the water/binder ratio must be above 0.78 for ye’elimite to fully react with 2 mol of anhydrite [6]. Table 9 summarizes representative EDS points to illustrate the main phases and the residual clinker regions observed in the SEM mortar images.

The analysis of the atomic ratios from Table 9 of the EDS test performed in the samples revealed the presence of unreacted/partially hydrated ye’elimite phases only for 50% and 75% CSA content, points P8; P9 and P12; P14, respectively. The lack of unreacted ye’elimite in the 25% CSA mixture suggested it was faster to react than the calcium silicate phases from PC with the available water and calcium sulphate. Additionally, P4, P10 and P11 indicated the presence of unreacted fly ash particles in the mixtures but particularly more abundant in the 25% CSA sample, in agreement with higher PC content and lack of fly ash reactivity at this young age. EDS also pointed to higher presence of unreacted PC particles in the mixtures with 75% CSA, which can suggest the more sulphate and water uptake from fast reacting ye’elimite particles, in agreement with the results from isothermal calorimetry and XRD tests. Ca-Si-rich regions with Ca/Si atomic ratios between 1.2 and 2.4 were detected in all mixtures and were attributed to C-(A)-S-H and partially hydrated calcium silicate clinker. C-(A)-S-H rich regions, visible in green boxes in Figure 13 and for points P1 and P6, indicated sulphate consumption to produce the Ca-Al-Si rich phases from ye’elimite and calcium silicates hydration. Overall, a more densely filled microstructure was found from 25 to 75% CSA, supporting the increase in compressive strength results with higher CSA content.

## 4. Conclusions

This study evaluated the effects of full and partial replacement of PC with CSA cement on setting times, workability, hydration, microstructure, and mechanical properties of pastes and mortars.

Increasing the CSA cement content tended to shorten the setting times of hybrid mixes. The statistical interaction between the w/b ratio and CSA cement content variables exhibited a setting time increase with higher w/b ratio only at low CSA cement content but this effect disappeared at higher CSA cement replacement percentages.

Higher CSA cement content decreased the workability of the PC-CSA mixes. Though the statistical interaction of the w/b ratio and CSA% was only significant at the 10-min slump test, which suggested a time-dependent effect and represented slump variations at 25 and 50% CSA with w/b ratio changes but virtually no slump at 75%, regardless of the w/b ratio.

The workability retention time depended on the quantity of added CSA cement. For 25% CSA mixes, workability could be retained for more than 30 min after mixing. Mixes with 50% CSA did not retain more than 20 min of open time and mixes with 75% only had flowability for the test done 2 min after the initial mixing.

Isothermal calorimetry indicated that the heat-flow behavior and cumulative heat of the mixes containing 50% and 75% CSA behaved closer to that of pure CSA cement In contrast, the 25% CSA mixture displayed hydration features associated with both portland cement and CSA cement.

CSA cement content consistently increased compressive strength at all tested curing, while w/b ratio generally reduced compressive strength though this was only statistically significant at 2 h. CSA content was the main factor governing compressive strength development while the influence of the w/b ratio depended on the CSA content and curing age.

Increasing CSA cement content generally improved flexural strength, particularly at 2 h while at 1 day this effect was not linear. CSA% was not statistically significant at 7 days for flexural strength while strength at this curing age was dominated by the influence of w/b ratio. W/b ratio proved a consistent negative effect on flexural strength at all ages.

XRD and FTIR analyses provided evidence of increased ettringite and C-S-H/C-(A)-S-H hydration products in the 50–75% CSA systems. SEM images evaluation and EDS spot analysis supported the development of a more densely filled microstructure with increasing CSA cement content. Microstructural and phase analysis was consistent with the observed enhancement in compressive strength. Nevertheless, a CSA content of 25% resulted in consistent strength development over the studied curing time for all the w/b ratios.

Overall, the findings underline the potential of hybrid PC-CSA mixes with 25% CSA in providing the most balanced performance, from early strength improvements while minimizing potential workability drawbacks associated with higher CSA contents.

This study represents an approach into the interaction of two mixture design parameters of cementitious blends. The findings show that depending on the CSA cement content, w/b ratio changes do not result in noticeable effects on the workability of the mixes. This highlights the need for admixtures to control and achieve the desired workability of the mixtures. Though the interaction effect is not present in the hardened state of the mixtures, both design parameters still affect their development. Therefore, this study shows the potential for high CSA cement content to be under special requirements, such as in sprayed concrete applications, where rapid strength development is required, through a tailored mix design while controlling to maintain the workability window.

## 5. Limitations and Future Work

While the findings of this study indicate its potential, a limitation of this study is that it focused on one kind of CSA cement; hence, it is recommended that future research focuses on a wider range of this cement. Additionally, the long-term effect of high CSA cement content should be further evaluated along with its effect on shrinkage, durability and its environmental impact for a more thorough understanding of its suitability as a portland cement partial replacement.

Even though the microstructure of the mortars was studied via backscattered mode in polished samples, using secondary electron mode on raw mortar surfaces at higher magnification could allow to explore properly the morphology of the hydrated samples and even explore microcracks that might be linked to strength decreases at 7 days and later ages.

While the heat of hydration of the blended cement pastes was explored in this study, the use of external mixing might lead to an inaccurate representation of the first minutes of heat development. Therefore, the use of internal mixing is recommended when studying CSA cement.

The characteristics of the studied pastes and mortars mixes cannot be directly used to evaluate concrete, as the presence of bigger aggregates in the mixtures will affect the expansive behavior of the CSA cement. Thorough evaluation of concretes made with the studied ranges is encouraged.

## Figures and Tables

**Table 1:** Main characteristics of PC and CSA cement (according to the manufacturers).

	Density [kg/m^3^]	Blaine Specific Surface Area [m^2^/kg]	Anhydrite Content (%)	C_4_A_3_$\bar{S}$ Content (%)	Fly Ash Content (%)
PC	3000	430	-	-	14
CSA cement	2800	>400	19	45	-

**Table 2:** XRF oxide composition of CSA cement and PC.

Chemical Composition (%)	CSA Cement	PC
SiO_2_	6.80	18.95
Al_2_O_3_	24.33	3.51
Fe_2_O_3_	1.42	5.15
CaO	43.97	62.96
MgO	0.84	1.54
SO_3_	17.80	3.56
K_2_O	0.18	0.73
TiO_2_	1.03	0.45
MnO_3_	0.02	0.09
SrO	0.10	0.05
BaO	0.03	0.03
Cr_2_O_3_	0.04	0.01
V_2_O_5_	0.02	0.02
ZnO	0.00	0.04
ZrO_2_	0.06	0.02
LOI 950 °C	1.79	2.83
Sum	98.51	96.85

**Table 3:** Paste mixture proportions per 500 g of binder and identifiers.

Mix ID	CSA%	PC (g)	CSA Cement (g)	w/b	SP (g)
PC25W35	25	375	125	0.35	1.0
PC25W385	25	375	125	0.385	1.0
PC25W42	25	375	125	0.42	1.0
PC50W35	50	250	250	0.35	1.0
PC50W385	50	250	250	0.385	1.0
PC50W42	50	250	250	0.42	1.0
PC75W35	75	125	375	0.35	1.0
PC75W385	75	125	375	0.385	1.0
PC75W42	75	125	375	0.42	1.0

**Table 4:** Mortar mixture proportions and identifiers.

Mix ID	CSA%	PC (kg/m^3^)	CSA Cement (kg/m^3^)	w/b	Sand (kg/m^3^)	SP (kg/m^3^)
MC25W42	25	300	100	0.42	800	0.4
MC25W47	25	300	100	0.47	800	0.4
MC25W52	25	300	100	0.52	800	0.4
MC50W42	50	200	200	0.42	800	0.4
MC50W47	50	200	200	0.47	800	0.4
MC50W52	50	200	200	0.52	800	0.4
MC75W42	75	100	300	0.42	800	0.4
MC75W47	75	100	300	0.47	800	0.4
MC75W52	75	100	300	0.52	800	0.4

**Table 5:** Statistical analysis summary of the responses.

Response	Adj. R^2^	Analysis *p*-Value					
		A-CSA%	B-w/b Ratio	A × B	A^2^	B^2^	LoF
Initial setting time	0.9846	−, <0.0001	+, 0.0004	−, 0.0249	NS	NS	0.2623
Final setting time	0.9892	−, <0.0001	+, <0.0001	−, 0.0313	+, 0.0048	NS	0.6353
2 min slump	0.8414	−, 0.0201	+, 0.0002	NS	−, 0.0293	NS	0.1815
10 min slump	0.9243	−, <0.0001	+, 0.0016	−, 0.0191	−, <0.0006	NS	0.0603
2 h compressive strength	0.976	+, <0.0001	−, 0.0002	NS	NS	NS	0.201
1-day compressive strength	0.977	+, <0.0001	−, 0.0667	NS	−, <0.0001	−, 0.0140	0.056
7-day compressive strength	0.517	+, <0.0049	−, 0.1965	NS	NS	NS	0.56
2 h flexural strength	0.945	+, <0.0001	−, 0.0034	NS	NS	NS	0.382
1-day flexural strength	0.964	+, <0.0001	−, 0.0366	NS	−, 0.0005	NS	0.713
7-day flexural strength	0.39	+, 0.7153	−, 0.0236	NS	NS	NS	0.369

**Table 6:** Heat of hydration descriptors of tested mixes.

Mix ID	Induction (h)	t_peak_ (h)	q_peak_ (mW/g)	Q_24_ (J/g)	Q_72_ (J/g)	Q_168_ (J/g)
PC	2.69	13.30	2.55	173.73	278.27	315.52
CSA25%	0.43	0.86	28.87	146.34	268.27	313.02
CSA50%	0.23	0.38	44.38	230.26	264.91	286.06
CSA75%	0	0.12	87.47	209.88	221.69	230.48
CSA100%	0.05	0.09	56.04	243.42	252.72	258.69

**Table 7:** Phase quantification of the blended cement pastes.

Phase	CSA25%	CSA50%	CSA75%
Ettringite	25.2 ± 3	33.7 ± 4	35.1 ± 2
Belite	18.3 ± 4	22.4 ± 5	22.7 ± 4
Alite	18.5 ± 2	20.2 ± 3	15.3 ± 3
Ye’elimite	1.9 ± 1	4.2 ± 2	11.0 ± 2
Calcite	6.1 ± 2	1.1 ± 0.9	0.8 ± 0.2
Hemicarboaluminate	4.1 ± 0.5	4.0 ± 1	0.5 ± 0.2
Portlandite	2.2 ± 0.9	1.0 ± 0.5	1.6 ± 0.8
Gypsum	0.7 ± 0.3	0.2 ± 0.1	0.4 ± 0.1
Quartz	0.8 ± 0.1	0.6 ± 0.3	0.3 ± 0.1
Amorphous	22.2 ± 4	12.6 ± 5	12.4 ± 2
GOF	1.35	1.4	1.3
RwP	6.95	7.4	6.77

**Table 8:** Compressive and flexural strength at early ages (mean ± SD).

Mix ID	Compressive Strength (MPa)			Flexural Strength (MPa)		
	2 h	1-Day	7-Day	2 h	1-Day	7-Day
MC25W42	13.4 ± 0.1	17.3 ± 0.9	49.1 ± 2.4	3.5 ± 0.1	5.6 ± 0.7	13.9 ± 0.6
MC25W47	8.7 ± 0.3	13.6 ± 0.3	53.6 ± 0.1	2.8 ± 0.1	4.9 ± 0.1	10.4 ± 0.9
MC25W52	6.3 ± 0.4	9.2 ± 0.5	50.3 ± 4.0	2.3 ± 0.1	2.9 ± 0.2	10.4 ± 0.4
MC50W42	27.1 ± 2.1	55.8 ± 3.9	81.4 ± 4.5	8.7 ± 0.1	11.2 ± 0	13.3 ± 0.7
MC50W47	22.3 ± 1.0	59.0 ± 1.9	59.2 ± 5.6	7.5 ± 0.4	12.0 ± 0.6	12.1 ± 1.2
MC50W52	21.1 ± 1.0	52.5 ± 1.7	56.8 ± 4.2	6.1 ± 0.3	10.9 ± 1.1	10.4 ± 0.4
MC75W42	41.2 ± 2.1	63.7 ± 9.6	75.9 ± 8.8	10.6 ± 0.3	13.3 ± 1.4	11.7 ± 0.5
MC75W47	41.3 ± 1.1	73.6 ± 3.4	70.3 ± 8.4	10.3 ± 1.2	13.3 ± 2.2	12.3 ± 1.0
MC75W52	31.5 ± 0.9	58.1 ± 1.1	73.6 ± 4.5	8.6 ± 0.5	7.9 ± 0.6	11.6 ± 1.0

**Table 9:** Representative atomic ratios of PC-CSA mixtures.

CSA Content	EDS Point	Ca/Si	Al/Si	Ca/Al	S/Al	Ca/S
25%	P1	3.43	2.03	1.69	0.16	6.13
25%	P2	1.26	0.13	10.09	0.26	3.89
25%	P3	2.35	1.57	1.49	0.09	11.05
25%	P4	0.07	0.10	0.68	-	-
25%	P5	1.61	0.71	2.25	0.36	2.74
50%	P6	1.17	0.74	1.59	0.27	3.71
50%	P7	1.41	0.15	9.68	0.33	3.06
50%	P8	1.37	0.62	2.20	0.17	5.79
50%	P9	2.46	4.71	0.52	0.16	6.17
50%	P10	0.06	0.11	0.59	-	-
75%	P11	0.12	0.73	0.16	0.05	3.35
75%	P12	1.38	1.92	0.72	0.19	3.73
75%	P13	1.56	0.20	7.69	0.19	40.47
75%	P14	2.37	1.80	1.32	0.55	2.38

## Data Availability

The original contributions presented in this study are included in the article. Further inquiries can be directed at the corresponding author.
